# Supplementary material for: An Interaction Network of RNA-Binding Proteins Involved in Drosophila Oogenesis
Source: Mol Cell Proteomics. 2020 Nov 25;19(9):1485–502. doi: 10.1074/mcp.RA119.001912 (PMC8143644; doi:10.1074/mcp.RA119.001912)

| Raw file | Scan | Method | Score | m/z |
|---|---|---|---|---|
| 20170411_CO_0685FuBo_R17R18R19_Mix1 | 6898 | FTMS; HCD | 164.93 | 794.41 |

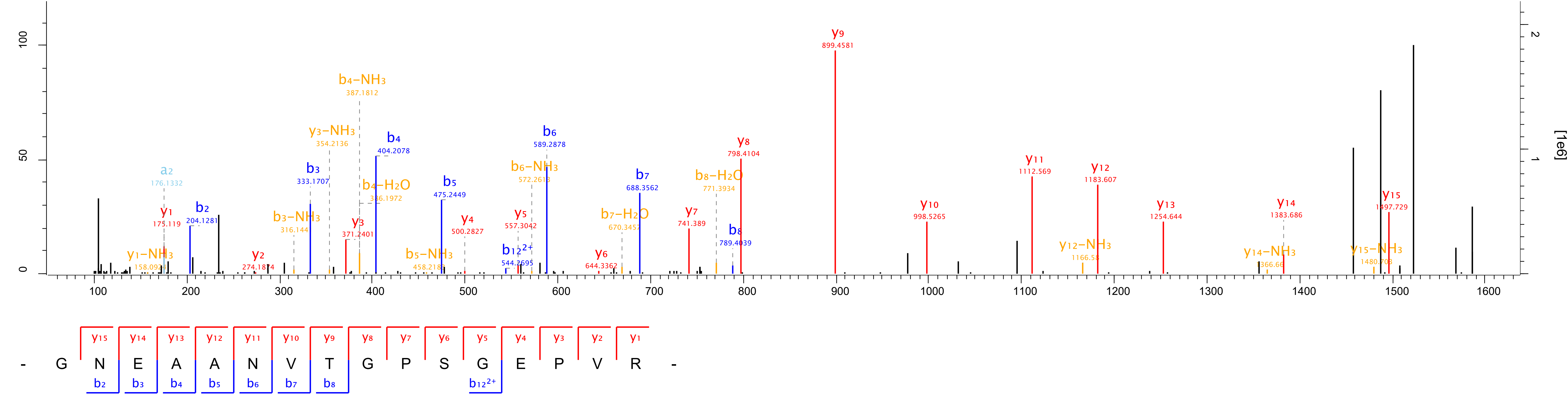

Supplement: Supplementary file 1 [file mmc1.zip › mmc1/157750_1_supp_536530_qry2q7.zip!/Single_peptide_identifications_R17-R22/20170411_CO_0685FuBo_R17R18R19_Mix1_6898.pdf]
